# Direct visualization of the extracellular binding structure of E-cadherins in liquid

**DOI:** 10.1038/s41598-020-72517-2

**Published:** 2020-10-12

**Authors:** Teiko Shibata-Seki, Masato Nagaoka, Mitsuaki Goto, Eiry Kobatake, Toshihiro Akaike

**Affiliations:** 1 Biomaterials Center for Regenerative Medical Engineering, Foundation for Advancement of International Science, 24-16 Kasuga, 3-chome, Tsukuba, Ibaraki 305-0821 Japan; 2 School of Life Science and Technology, Tokyo Institute of Technology, G1-13, 4259, Nagatsuta, Midori-ku, Yokohama, Kanagawa 226-8502 Japan

**Keywords:** Cadherins, Atomic force microscopy

## Abstract

E-cadherin is a key Ca-dependent cell adhesion molecule, which is expressed on many cell surfaces and involved in cell morphogenesis, embryonic development, EMT, etc. The fusion protein E-cad-Fc consists of the extracellular domain of E-cadherin and the IgG Fc domain. On plates coated with this chimeric protein, ES/iPS cells are cultivated particularly well and induced to differentiate. The cells adhere to the plate via E-cad-Fc in the presence of Ca^2+^ and detach by a chelating agent. For the purpose of clarifying the structures of E-cad-Fc in the presence and absence of Ca^2+^, we analyzed the molecular structure of E-cad-Fc by AFM in liquid. Our AFM observations revealed a rod-like structure of the entire extracellular domain of E-cad-Fc in the presence of Ca^2+^ as well as *trans*-binding of E-cad-Fc with adjacent molecules, which may be the first, direct confirmation of *trans*-dimerization of E-cadherin. The observed structures were in good agreement with an X-ray crystallographic model. Furthermore, we succeeded in visualizing the changes in the rod-like structure of the EC domains with and without calcium. The biomatrix surface plays an important role in cell culture, so the analysis of its structure and function may help promote cell engineering based on cell recognition.

## Introduction

**Figure 1:**
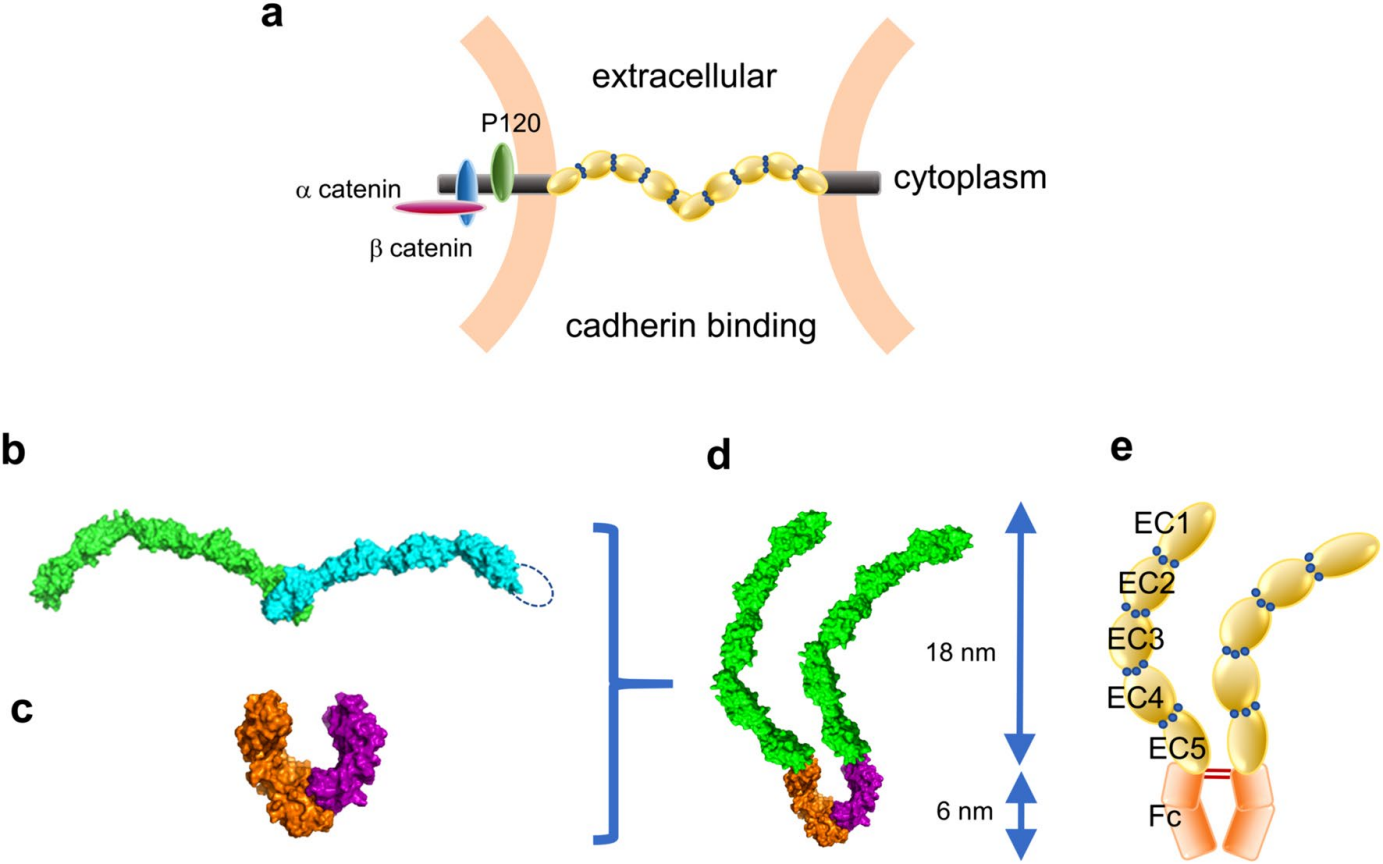
E-cadherin model. (**a**) Schematic illustration of E-cadherin binding. The extracellular site of E-cadherin is composed of five domains bound in tandem. The binding model is based on crystal structure (PDB ID: 3Q2V^28^). (**b**) Crystallography structural model (PDB ID: 3Q2V^28^) of E-cadherin extracellular domains created with PyMOL. (**c**) Crystallography structural model (PDB ID: 5JII^44^) of IgG-Fc domain created with PyMOL. (**d**) E-cadherin-Fc molecular model created by combining two crystallography structural model (**b**,**c**). (**e**) Schematic illustration of E-cadherin-Fc molecular model. EC5 domain is connected to Fc fragment. Calcium ions are shown as blue spheres.

Cadherin is a group of cell surface glycoproteins, identified and named as a calcium-dependent cell adhesion molecule^1^. There are two types of cell adhesion, cell–cell adhesion and cell-substrate adhesion. Cadherin forms the former type of homophilic adhesion. There are more than 100 types of cadherins, among which classical cadherins are the most studied. Classical cadherins are single-pass transmembrane proteins of the cell membrane. On the cytoplasmic side, cadherins link to actin cytoskeleton through such molecules as catenin groups and EPLIN. Many physiological studies have been carried out to show that cell–cell adhesion is controlled from the cytoplasmic side^2–6^. The extracellular site is composed of five domains bound in tandem (Fig. 1a), and the size of one domain is about 110 amino acid residues, which are named Extra Cellular 1 (EC1) -EC5 in order from the N-terminal side. Cadherins bind by binding calcium ions to calcium ion binding sites between individual extracellular domains. The EC1 and EC2 domains are the ones directly involved in the cell adhesion, and there have been many studies on the interactions between them.

E-cadherin-Fc (E-cad-Fc) is a fusion protein, which consists of the extracellular domain of E-cadherin and the IgG Fc domain^7–10^ (Fig. 1e). E-cadherin involves in cell morphogenesis, embryonic development, cancer, EMT, etc.^11–15^ so that the chimeric proteins may serve as important biomaterial which helps clarify the cell function. On plates coated with E-cad-Fc, ES/iPS cells are cultivated particularly well and induced to differentiate with several methods^16–19^. Application of E-cad-Fc to particle coating is also in progress^20^. Chimeric proteins of the same kind, namely N-cad-Fc and VE-cad-Fc, have been also developed, which also promote differentiation of neural progenitor cells^21–24^ and human umbilical vein endothelial cells (HUVECs)^25^ respectively in vitro. Experiments in mice aimed at developing treatments for newborn brain injury showed that N-cad-Fc-coated gelatin sponge artificial scaffolds promote neuronal regeneration and functional recovery, highlighting the significance of biomaterials also in vivo^26^. Cells cultivated on plates coated with these cadherin chimeric proteins adhere in the presence of Ca^2+^ and detach when Ca^2+^ is removed by a chelating agent such as EDTA and EGTA. It is of great importance to clarify structures of the chimeric proteins in the presence and absence of calcium ions, as well as their cell adhesion mechanism, so that the chimeric proteins may be better used for biomaterial development.

With these backgrounds, we focus on morphological observations of E-cad-Fc by using AFM (Atomic Force Microscope). AFM allows direct observation of a sample as it is. Unlike an electron microscope, samples do not need to be dried or frozen, and can be measured in a physiological environment. Furthermore, there is no need for sample staining. With AFM observation, a resolution of nm level is expected. For example, the periodic double-helix structure of B-DNA in the solution was observed at a spatial resolution of 1.2 nm with the PeakForce Tapping mode^27^. The short axis diameter of the EC domain is expected to be around 2 nm (calculated from PDB ID: 3Q2V^28^, Fig. 3b), so that AFM may be well suited for our study to capture E-cad-Fc structure in cell culture.

Since cadherins are membrane protein and tend to aggregate and are difficult to analyze as a whole molecule, previous analyses were limited to their hydrophilic EC domains. To the best of authors’ knowledge, the first AFM observation of cadherins in liquid was performed by Baumgartner et al.^29^ on VE cadherin which belongs to the type II classical cadherin family. Later, Brasch et al*.*^30^*.* obtained AFM images of molecules under ambient condition which are presumed to be monomers and dimers of VE cadherin. Harrison et al*.*^31^ made an AFM observation in liquid for the entire extracellular domain of N-cadherin which belongs to the type I classical cadherin family. For our observations, E-cad-Fc is employed, which is soluble and easy to handle. E-cad-Fc molecules in solution may take two states: in one state, two EC domains within the same E-cad-Fc molecule interact laterally, while the EC domain *trans*-interacts with different E-cad-Fc molecules in the other state. In other words, there is a possibility that both *cis* and *trans* dimerization may occur. Although there had been much debate on which dimer, namely *cis* or *trans*-dimer^32–37^, forms initially, various studies over the past decades have established that *trans* dimerization precedes *cis*-dimer formation^28,38–43^. The features observed here were *trans*-binding as reported.

In this paper, we report the results of direct AFM observations of E-cad-Fc under physiological conditions in vitro*.* Our AFM observations successfully visualize a structure of the entire extracellular domain of E-cad-Fc in the presence and absence of Ca^2+^, as well as molecules configuration which may be presumed as *trans*-binding of E-cad-Fc with adjacent molecules. The observed structures were in good agreement with an X-ray crystallographic model. This may be the first AFM observation of the entire extracellular domain and *trans*-binding of E-cadherin in the presence of Ca^2+^. Furthermore, dynamic shape changes of the EC domain with the addition of EDTA are also shown.

## Results and discussion

The molecular model shown in Fig. 1d was created by combining two molecular structures obtained by crystal structure analysis. Figure 1b is the structure in which the curved rod-like extracellular site of E-cadherin is *trans*-coupled as reported by Harrison et al*.* (PDB ID: 3Q2V^28^), and Fig. 1c is the structure of the Fc portion of IgG by Lobner et al*.* (PDB ID: 5JII^44^). EC domains are connected to hinge regions of Fc domains via linkers.

The adhesive capability of E-cad-Fc was reported by Nagaoka et al*.*^8–10^. EB3 mouse embryonic stem (ES) cells adhered to the E-cad-Fc coated surface with the same efficiency as the conventional gelatin coated surface, and this adhesion was Ca^2+^ dependent. Many applications using the recombinant E-cad-Fc were also reported^16–20,45^. We also examined the adhesive activity of cadherin using E-cad-Fc coated polystyrene beads. The results showed that the beads adhere to each other depending on Ca^2+^ concentration (Supplementary Data S1).

**Figure 2:**
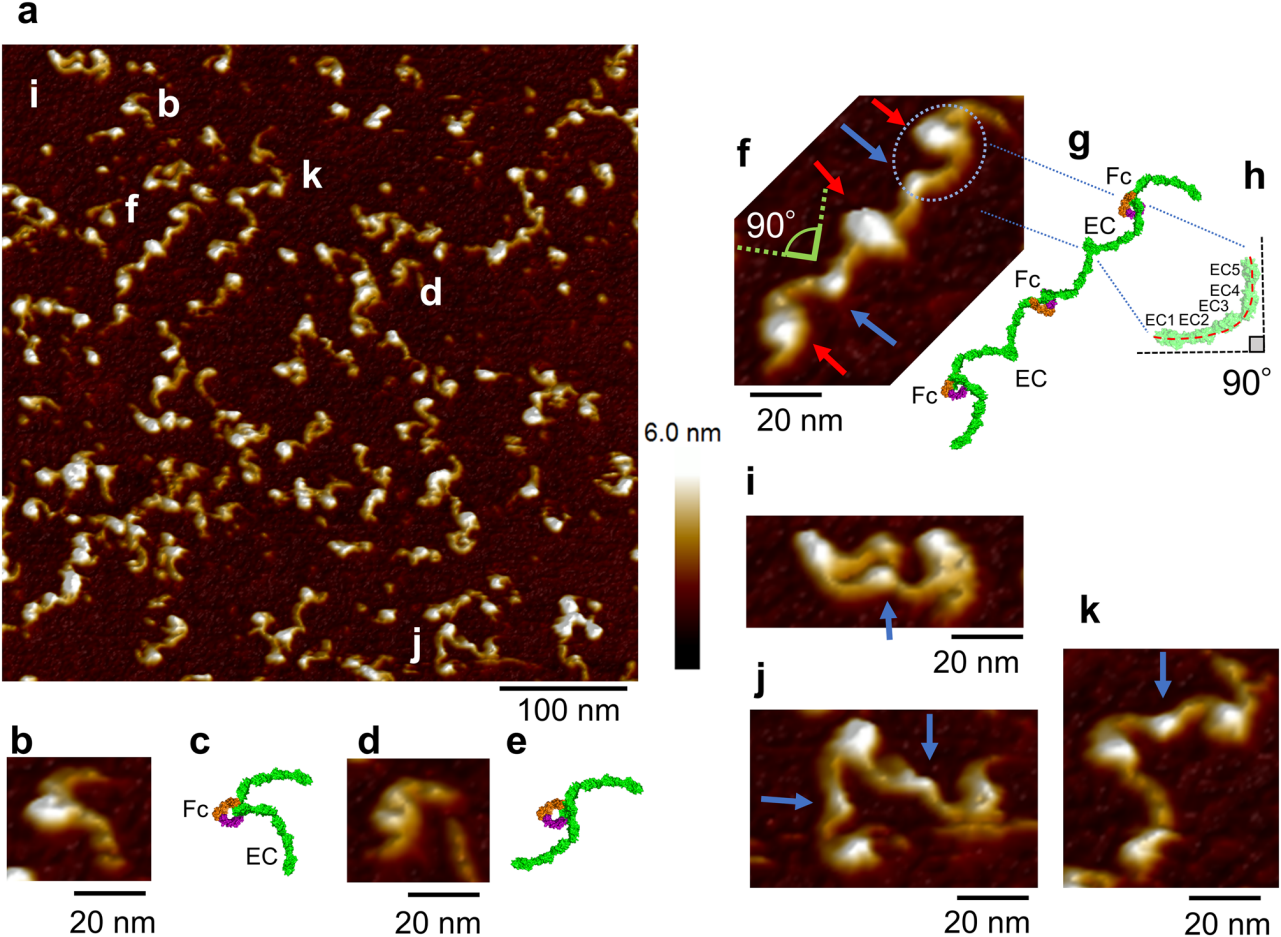
AFM images of E-cad-Fc molecules in a PBS solution containing 0.9 mM Ca^2+^ at room temperature and their structural models. (**a**) AFM image of E-cad-Fc molecules. E-cad-Fc molecules were observed in various conformations (scale bar 100 nm). (**b**,**d**) Magnified AFM images of E-cad-Fc monomer-like structures of the points b and d in (**a**) (scale bar 20 nm). (**c**,**e**) E-cad-Fc monomer models constructed from the AFM measurement results of (**b**) and (**d**). The orientation and tilt of the Fc domain are arbitrary due to insufficient resolution of AFM image. (**f**) Magnified AFM image of E-cad-Fc molecules of the point f in (**a**) (scale bar 20 nm). Three E-cad-Fc molecules appear *trans*-bonded (blue arrow) in the EC1 domain. EC domains (blue dotted circle) and Fc domains (red arrow) are distinguishable. (**g**) E-cad-Fc model constructed from the AFM measurement results of (**f**). (**h**) EC domain model. The five EC domains are curved such that the long axis of EC1 is approximately perpendicular to the long axis of EC5^46–48^. (**i**–**k**) Magnified AFM images of E-cad-Fc binding structures of the molecules i, j and k in (**a**) (scale bar 20 nm).

**Figure 3:**
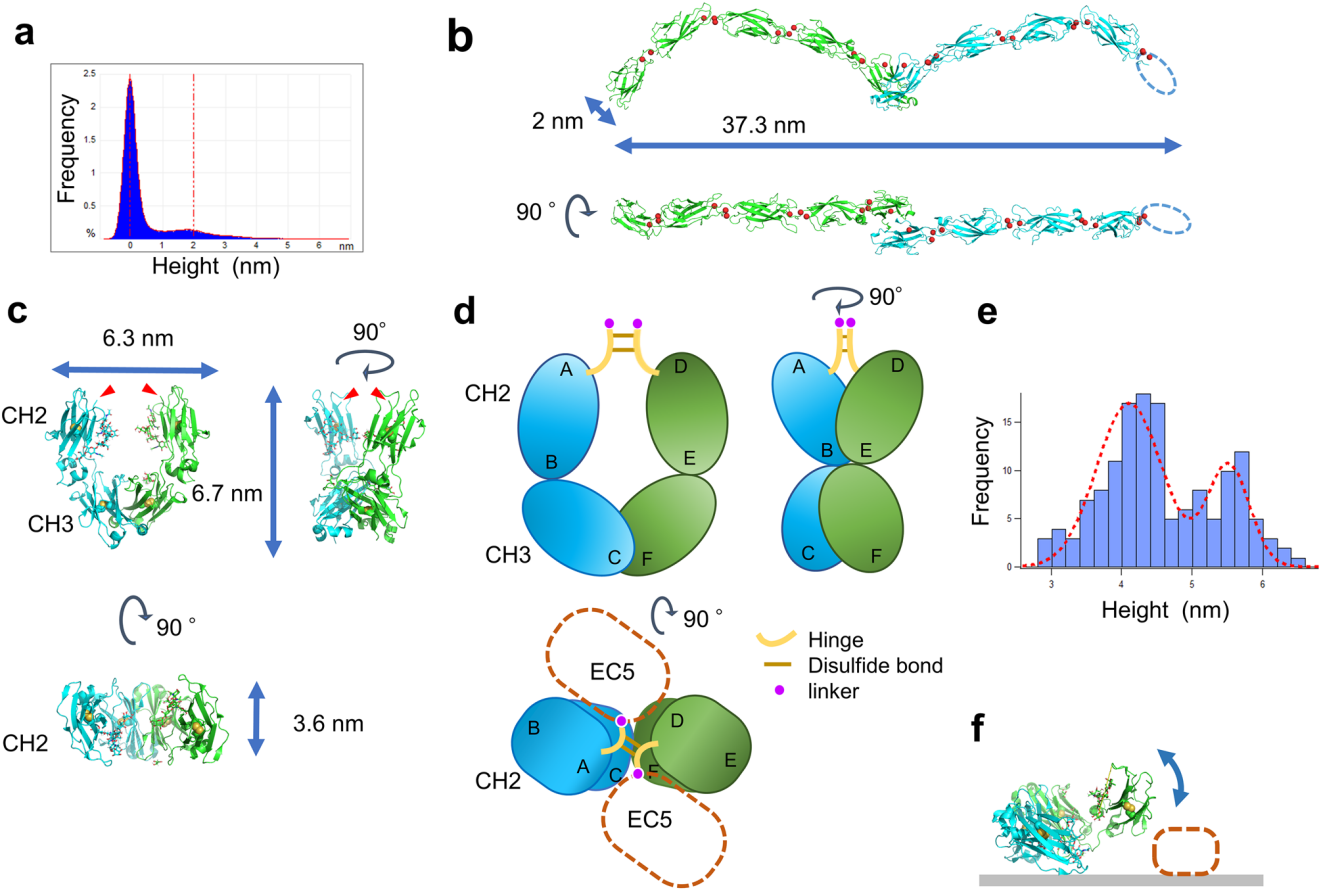
The related analyses of AFM images. (**a**) Height distribution of the AFM image Fig. 2a (n = 262,144). The peak-to-peak is 2.02 nm. (**b**) Ribbon style view of crystallography structural model of E-cadherin extracellular domains created with PyMOL. The EC5 of one side was not resolved (PDB ID: 3Q2V^28^) and is represented by a dotted line. Calcium ions are shown as red spheres. (**c**) Crystallography structural model (PDB ID: 5JII^44^) of IgG-Fc domain created with PyMOL. Hinge regions are not included in the model. The hinge region follows the N-terminal, residue 237^53^ (red triangular), which appears in this crystallography structural model. Examination with PyMOL revealed that the size of the Fc domain was 6.7 nm in length, 6.3 nm in width, and 3.6 nm in height. The three-dimensional structure is shown in movie in Supplementary Video S2. (**d**) Schematic illustration of Fc molecular model. The CH2CH3 chains are bent and in a skewed position. EC domains are connected to Fc domains comprising CH2, CH3 and hinge regions via linkers. Hinge regions are shown as yellow line. Linkers are shown as violet circle. Brown lines represent disulfide bonds. Because residue 237 is located on the inner periphery of the skewed U-shaped Fc domain, it is presumed the hinge and linker region between EC5 domain and CH2 is located in this neighborhood. (**e**) Height analysis of Fc domains. The distribution was constructed by using the height data obtained from locations which are considered to correspond to the Fc domain of the E-cad-Fc in Fig. 2a. The median and variance for the first peak were 4.1 nm ± 0.66, while those for the second peak were 5.5 nm ± 0.42 by Gaussian fitting (n = 148). (**f**) The tilted Fc domain “lying down” model.

Cells are cultured on E-cad-Fc coated polystyrene dishes, whereas AFM observation was performed on atomically flat mica. Mica was used after being made hydrophobic by treatment with poly-l-ornithine coat. The series of figures in Fig. 2 show AFM images of E-cad-Fc molecules in PBS buffer solution containing 0.9 mM Ca^2+^. Various conformational E-cad-Fc molecules are observed in the wide area image of Fig. 2a, with EC domains and Fc domains clearly distinguishable. Figure 2b,d are magnified views from Fig. 2a, which may be presumed an E-cad-Fc monomer, and Fig. 2c,e are molecular models constructed from these AFM images respectively. As will be described in detail below, the observed curvature and height of the entire EC domain are in good agreement with the x-ray structural model. Figure 2f is a magnified view from Fig. 2a, which may be presumed three E-cad-Fc molecules, and Fig. 2g is a molecular model constructed from these AFM images. The comparison with the molecular model by crystal structure analysis Fig. 3b suggests that three E-cad-Fc molecules are *trans*-bonded in the EC1 domain, where the following three points are of particular interest.

1. The curvature of the rod-like structure of EC domain (Fig. 2f, the blue dotted circle).
  The curvature of the EC domains observed with AFM are similar to that found in the crystal structure (Fig. 3b). The crystal structure analysis^46^ and the electron micrographs observation^47,48^ reported that the five EC domains are curved such that the long axis of EC1 is approximately perpendicular to the long axis of EC5 (Fig. 2h).
2. The angle of the EC1-binding part (Fig. 2f, the blue arrow).
  The angle between EC1-binding part was measured to be about 90° in our AFM image, which coincides very well with the previous results of ~ 88° by Harrison et al.^28^ and Häussinger et al*.*^49^.
3. The measured size of the rod-like structure of EC domain.
  When the crystal structure data are examined with the structural analysis PyMOL software (version 2.0.4)^50^ (Schrödinger, Inc., USA), the short axis diameter of EC is about 2 nm, with the length in the major axis direction being about 18 nm (Fig. 3b). According to the analysis of Harrison et al*.*^28^, the distance between the C-termini of the *trans*-dimer was 37.3 nm. The AFM image of Fig. 2f gives the results that the diameter measured by height is 2 nm, and the distance between C-terminals is about 35 nm. When measuring a cylindrical diameter by AFM, the height (Z) information was employed instead of the X–Y plane information, because the X–Y plane measurement includes a convolution effect with the cantilever tip^51,52^. Since the C-terminus overlaps with the Fc domain and the position cannot be determined accurately, it is likely that the value is slightly smaller than that obtained by the crystal structure analysis. The height of the overlapping part of the EC1 (Fig. 2f blue arrow) of the dimer was measured to be about 4 nm. In Fig. 2a, ten locations were identified where the overlapping of the EC1 could be clearly recognized, and the average height of those locations was found to be 4.0 (0.27) nm (mean (SD)). These are in good agreement with the X-ray crystallographic model examined by PyMOL.
  The height of the EC domain was calculated as the height difference between the two peaks observed in the height distribution (Fig. 3a) of the AFM image (Fig. 2a). The calculation by using the software bundled to the AFM instrument. The height distribution was obtained from the height data at the 262,144 (= 512 × 512) locations in the image, where the peak location of the largest frequency is considered as the substrate portion and the other peak location is deemed as the EC domain. The calculated EC domain height was 2.02 nm, which coincides very well with that obtained from the crystal structure analysis.

These results imply that observed structures are *trans*-bonded E-cad-Fc molecules in the EC1 domain. Some of the obvious *trans*-bonded E-cad-Fc molecules in Fig. 2a are shown as magnified views in Fig. 2i–k where their EC1-binding parts are pointed with blue arrows.

Regarding the height of the Fc domain, two peaks were found in its height distribution as shown in Fig. 3e. The distribution was constructed by using the height data obtained from locations which are considered to correspond to the Fc domain of the E-cad-Fc in Fig. 2a. The followings were excluded from the height distribution construction: the overlapping portion of the EC1, the portion where no connection with the EC1 was observed, the aggregate, and the portion where the measurement was corrupted with noise. The heights of the two peaks were obtained by Gaussian fitting using IGOR Pro software (version 6.3.6). The median and variance for the first peak were 4.1 nm ± 0.66, while those for the second peak were 5.5 nm ± 0.42 (n = 148). Examination with PyMOL revealed that the size of the Fc domain was 6.7 nm in length, 6.3 nm in width, and 3.6 nm in height (Fig. 3c).

It is unknown which part of the Fc domain is in contact with the mica on substrate. Judging from the shape of the molecular model, it is likely that the widest side of the Fc is “lying down” in contact with the mica. If this is the case, the height of the Fc domain measured by the AFM should be around 3.6 nm (Fig. 3c), but this value does not match the measurement result. The Fc domain consists of two CH2CH3 chains, which are the constant regions of an immunoglobulin heavy chain. The two CH2CH3 chains are bent and in a skewed position, rather than on the same plane (Supplementary Video S2). Hinge regions are not included in this crystallography structural model. The hinge region follows the N-terminal, residue 237^53^, in this model. The two EC domains are connected via linkers to Fc domains comprising CH2, CH3 and hinge regions. Because residue 237 is located on the inner periphery of the skewed U-shaped Fc domain, it is presumed the hinge and linker region between EC5 domain and CH2 is located in this neighborhood (Fig. 3d). Therefore, the EC5 domains may tilt Fc domains which are ‘lying down’. If Fc domains are tilted, the height will be more than 3.6 nm. Our AFM measurements revealed two peaks at 4.1 nm and 5.5 nm in the height distribution for the Fc domain (Fig. 3e), which are larger than 3.6 nm. These results support the assumption that Fc domains are tilted. Further if EC5 domain wedges in between the CH2CH3 chain and the mica substrate, the height of Fc will be 5.6 nm at maximum (Fig. 3f).

These measurements have been achieved with a very weak load of 60 pN. This has been made possible by the excellent performance of the device and the carefully prepared cantilever.　The PeakForce Tapping mode controls the load on the sample by the movement of the piezo without using the resonance frequency of the cantilever, which improves the operability in liquids where the measurement is usually more challenging^54–57^. In the case of a soft sample, the cantilever tip may deform the sample if the load is too large. In the DNA measurement example, it was reported that the difference in loading force causes a difference in DNA height^27^. To keep the load as small as possible, we used a cantilever with an extremely low spring constant of 0.12 Nm^−1^. Since our measured EC height of E-cad-Fc measured by AFM was around 2 nm, which is consistent with the crystal structure analysis, the loading force of 60 pN was small enough not to give a significant deformation to the sample. Since the measurement was performed in solution, there is a possibility that hydration water around the sample and the electric double layer may affect the measurement^58^. To evaluate the absolute value of the height measurements, it is necessary to consider these influences.

**Figure 4:**
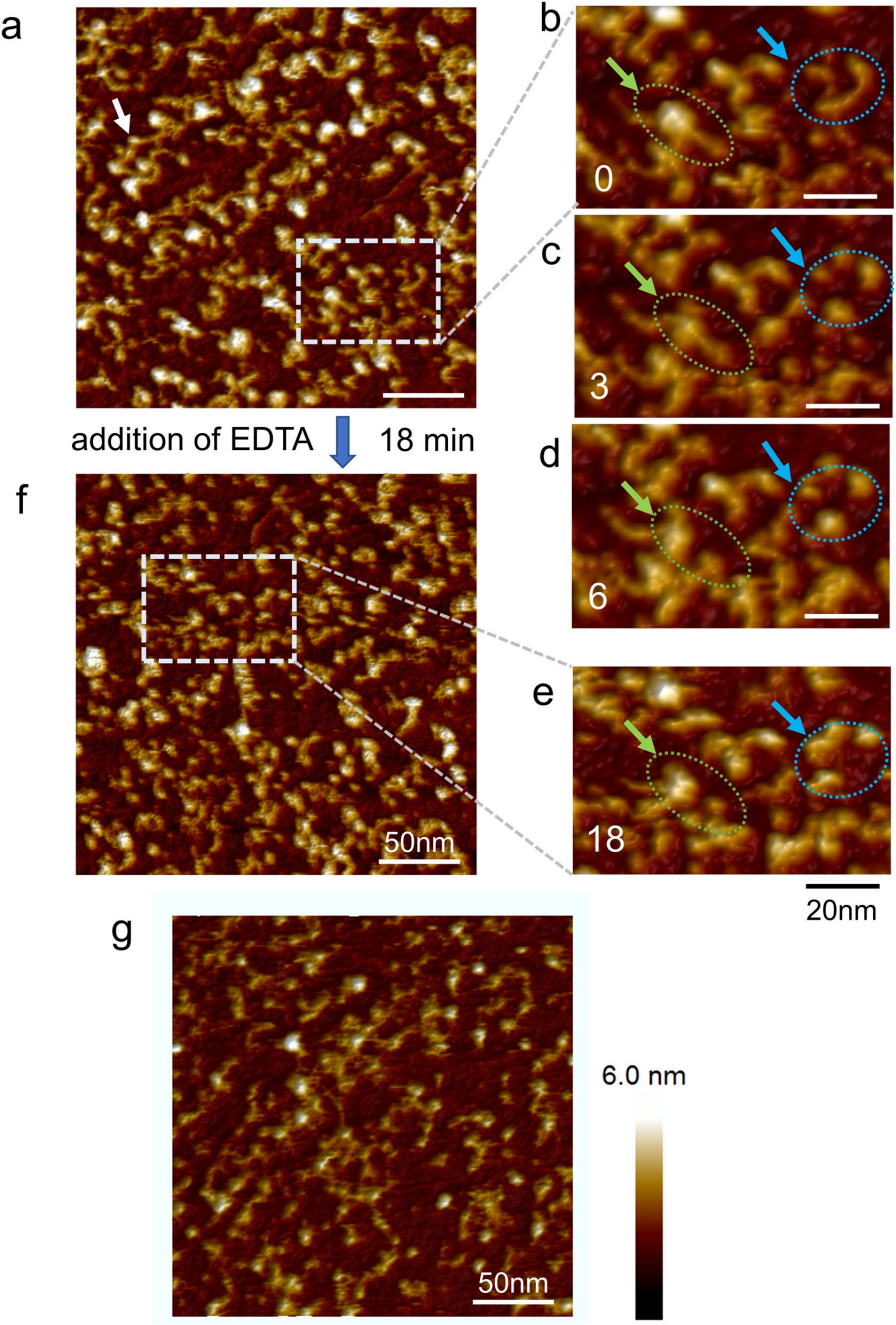
AFM images of the dynamic changes E-cad-Fc. (**a**) AFM image of E-cad-Fc in a PBS solution containing 0.9 mM Ca^2+^ before the addition of chelating agent, EDTA (scale bar 50 nm). It shows an EC domain with a rod-like structure, a *trans-*binding portion of the EC1 (pointed by white arrow) and an Fc domain. (**b**)**–**(**d**) The continuously obtained AFM images about 0, 3, 6, 18 min after addition of EDTA (scale bar 20 nm). (**e**,**f**) AFM images of E-cad-Fc 18 min after addition of EDTA (scale bar 20 nm and 50 nm respectively). (**g**) AFM image of E-cad-Fc in a PBS solution without Ca^2+^(scale bar 50 nm).

In Fig. 4, a series of images for E-cad-Fc are shown when the concentration of Ca^2+^ is changed by adding chelating agent. Figure 4a is an AFM image in a PBS buffer which contains 0.9 mM Ca^2+^ before adding chelating agent. It shows an EC domain with a rod-like structure, a *trans*-binding portion of the EC1 (pointed by white arrow), and Fc domains. EDTA was added to the PBS buffer solution so that its final concentration is 5 mM. Figure 4b–e show magnified views from time-laps images of 0, 3, 6, 18 min after the addition of EDTA capturing dynamic changes of the molecules. It takes about 3 min to obtain one image which consists of 256 lines, with a scan range of 250 nm and a scan speed of 1.5 Hz.

Here we focus on the molecular structures at two locations in Fig. 4 which have shown clear morphology changes. The first one is the molecule within the green circle, which appears to be an Fc domain with a rod-like structure. In Fig. 4b,c, the rod-like structure can be clearly discerned, but in Fig. 4d which is the image for 6 min after the addition of EDTA, the rod-like structure seems to have disappeared. Our second focus is on the molecule within the blue circle. In Fig. 4b, two rod-like structures can be observed. But in Fig. 4c, the connections seem to have been severed. Figure 4f is an image obtained 18 min after the EDTA addition, where rod-like structures are almost invisible. In other words, the chelation effect was successfully visualized at the molecular level. Figure 4g shows an AFM image of E-cad-Fc in a PBS solution without Ca^2+^. It has the same shape as the image of E-cad-Fc in a PBS solution without Ca^2+^. Baumgartner et al*.*^29^ reported that AFM-observed VE-cad-Fc in a buffer lacking Ca^2+^ was globular or V-shaped, but we observed only globular shapes. In this study, it took 3 min to obtain one frame. With the high-speed AFM technology^59–63^, which is capable of imaging at 50–100 ms per frame under physiological conditions, more detailed dynamic observation could be possible.

In the AFM observations of VE-Cadherin-Fc in a buffer by Baumgartner et al*.*^29^, the spatial resolution was about 10 nm, while it was about 2–4 nm in our study. One of the key factors for our success in acquiring high-resolution images in liquid is the use of cantilevers whose tip is extremely sharp with the nominal radius being 1 nm. When the same E-cad-Fc sample was measured with another cantilever, whose nominal tip radius is 2 nm, the Fc and the EC domains could not be distinguished, with only a rod-like structure being observed (Supplementary Fig. S3). The resolution of an AFM image on the XY-plane depends on the radius of curvature at a cantilever tip. Specifically, an apparent width (W) becomes W = 4(Rr)^1/2^, where R is the radius of curvature of the cantilever tip and r is the radius of a sample which is assumed to have a circular cross section^51,52^ (Supplementary Fig. S4). If we assume the EC domain is cylindrical with the radius of its cross section being 1 nm, the apparent width is calculated as 4 nm for a cantilever tip of R = 1 nm, and 5.64 nm for a R = 2 nm tip. The half-width value measured from the cross-sectional images of the EC domains in Fig. 2a is 4.73 nm ± 0.83 (n = 10) with a 1 nm cantilever tip. For the data measured with a cantilever whose nominal tip radius is 2 nm, the half-width value becomes 6.39 nm ± 0.88 (n = 10). Of course, there should be a variation in each cantilever tip, but we can certainly obtain higher resolution images by using a cantilever which has a sharp tip with a smaller radius. Further, force regulating capability as small as 60 pN contributes greatly to the successful imaging.

Mica is a substrate commonly used for the sample fixation in AFM measurement because it is easy to obtain a cleavage plane that is clean and atomically flat. The mica surface as prepared is generally hydrophilic, which makes sample fixation in an aqueous solution difficult. However, the coating with poly-l-ornithine turns the surface positively charged and the immobilization of E-cad-Fc via the Fc domain on the hydrophobic surface in an aqueous solution is made possible through the hydrophobic interaction, but the details of interaction forces contributing to the fixation are unknown.

## Methods

### Sample preparation and substrate

E-cad-Fc’s were genetically engineered by using the previously reported method^10^ and were stored in PBS solution (FUJIFILM Wako Pure Chemical Corporation, Japan) with Ca^2+^. Muscovite mica (ALLIANCE Biosystems, Inc., Japan) was coated by poly-l-ornithine (FUJIFILM Wako Pure Chemical Corporation, Japan) as follows. A 30 µl droplet of 0.1% poly-l-ornithine solution was deposited onto a freshly cleaved mica substrate (12 mm × 12 mm). Thirty minutes later, the substrate was rinsed with distilled water through a filter (Millex-GV, 0.22 μm, Merck Millipore Ltd., MA, USA) and dried. The PBS solution with Ca^2+^ was prepared with D-PBS (−) powder (FUJIFILM Wako Pure Chemical Corporation, Japan) and D-PBS ( +) Preparation Reagent (NACALAI TESQUE, INC., Japan). It contains KCl 200 mg/L, NaCl 8000 mg/L, KH_2_PO_4_ 200 mg/L, Na_2_HPO_4_ (anhyd.) 1150 mg/L, CaCl_2_ 100 mg/L, MgCl_2_∙6H_2_O 100 mg/L. The molar concentration of calcium in PBS solution with Ca^2+^ is 0.9 mM. 10 μg/mL and 20 μg/mL E-cad-Fc solution was prepared in PBS solution with Ca^2+^. A 30 µl droplet of the 10 μg/mL E-cad-Fc solution was deposited onto the ornithine coated mica substrate. AFM measurements were performed without rinsing.

For the time laps measurement, 12.5 mM EDTA was prepared with EDTA 2Na. For the control experiment, a 30 µl droplet of 20 μg/mL E-cad-Fc in the PBS solution was deposited on poly-l-ornithine coated mica. Thirty minutes later, the substrate was rinsed with the PBS solution and was measured in the PBS solution.

### AFM measurement

AFM images were measured by a MultiMode 8 with NanoScope V controller (Bruker Corp., Billerica, MA, USA). PeakForce Tapping (PFT), which we used for the imaging and measurement of the adhesion force, enables us to obtain quantitative nanomechanical properties at a relatively high speed. In this study, we also used PeakForce QNM (PFQNM)^64^. PFQNM uses PFT mode. PFQNM provides us information on nanomechanical properties such as modulus and adhesion. This PFQNM imaging mode enables us to identify variations of material properties at high resolutions simultaneously with the imaging of sample topography. The samples were imaged in the PBS solution with Ca^2+^ at room temperature and captured in 512 × 512 data points for 500 nm scan area with a scan rate of 1 Hz. For the E-cad-Fc imaging (Fig. 2), the set point was chosen as 60 pN, and force-distance curves were recorded 20 nm (PF amplitude of 10 nm), at a frequency of 2 kHz, using sharp probes (PEAKFORCE-HIRS-F-B, Bruker Nano Inc., Goleta, CA, USA) with a spring constant of 0.12 Nm^-1^, a nominal resonant frequency of 100 kHz in air, a nominal tip radius of 1 nm and reflective gold coating cantilever. For the time laps imaging (Fig. 4), the samples were captured in 256 × 256 data points for 250 nm scan area with 1.2 ~ 1.5 Hz, the set point was about 200 pN, and force-distance curves were recorded 100 nm (PF amplitude of 50 nm) at a frequency of 2 kHz, using the same kind of the probe. For the control imaging(Fig. 4g), the set point was set to 700 pN, force-distance curve were recorded 60 nm (PF amplitude of 30 nm) at a frequency of 2 kHz, using the standard probe for PFT measurements (SCANASYST-EFLUID + , Bruker Nano Inc., Goleta, CA, USA) with a spring constant of 0.7 Nm^-1^, a nominal resonant frequency of 150 kHz in air, a nominal tip radius of 2 nm and reflective gold coating cantilever.

The images were mainly analyzed with the bundled software and the height distribution analysis was done by Gaussian fitting using standard function of IGOR Pro software (version 6.3.6) (HULINKS Inc., Japan).

In the time-lapse observation, the chelating agent solution was added using a syringe from the solution inlet of the holder for measuring in liquid. The solution was provided through an appropriately sized silicone tube which was placed between the syringe and its inlet, rather than directly from the syringe, so that the solution injection may not disturb the measurement.

### Molecular structure analyses

We used the free available PyMOL software (version 2.2)^50^ for the molecular structure analyses. Standard functions such as image rotation, spatial distance measurement and movie editing were utilized for the analyses.

## Conclusion

In this paper, E-cad-Fc molecules were visualized in liquid by AFM. Particularly, the AFM images we have provided may be the first, direct confirmation of *trans*-dimerization for E-cadherin occurring independent of *cis*-dimerization. The EC binding structure was similar to the one obtained in the crystal structure analysis. Moreover, the shape changes during the addition of chelating agent were clearly captured.

The key to the successful observation may be attributed to the use of Fc bounded to the cadherin EC domain, which facilitates the fixation of the EC domain to the substrate and helps distinguish its orientation identifying its N-terminus. Also, the AFM’s capability of high resolution imaging of biological samples in liquid has been crucial.

The cells adhere to the plate via E-cad-Fc in the presence of Ca^2+^ and detach when Ca^2+^ is removed by a chelating agent. The corresponding morphological changes of E-cad-Fc as well as *trans*-binding of cadherins with adjacent molecules were successfully observed at molecular level. It should be noted these observations were made in liquid Ca^2+^ concentration of 0.9 mM which is the same as that for cell cultivation. Clarification of the structure and function of biomatrix molecules play crucial role in future application development in cell engineering.

## Supplementary information

Supplementary file 1

## References

[1] Yoshida-Noro C, Suzuki N, Takeichi M (1984). Molecular nature of the calcium-dependent cell-cell adhesion system in mouse teratocarcinoma and embryonic cells studied with a monoclonal antibody. Dev. Biol..

[2] Nelson WJ (2008). Regulation of cell-cell adhesion by the cadherin-catenin complex. Biochem. Soc. Trans..

[3] Weis WI, Nelson WJ (2006). Re-solving the cadherin-catenin-actin conundrum. J. Biol. Chem..

[4] Maul RS (2003). EPLIN regulates actin dynamics by cross-linking and stabilizing filaments. J. Cell Biol..

[5] Takeichi M (1988). The cadherins: cell-cell adhesion molecules controlling animal morphogenesis. Development.

[6] Takeichi M (2014). Dynamic contacts: rearranging adherens junctions to drive epithelial remodelling. Nat. Rev. Mol. Cell Biol..

[7] Higgins JMG (1998). Direct and regulated interaction of integrin αEβ7 with E-cadherin. J. Cell Biol..

[8] Nagaoka M, Ise H, Akaike T (2002). Immobilized E-cadherin model can enhance cell attachment and differentiation of primary hepatocytes but not proliferation. Biotech. Lett..

[9] Nagaoka M (2006). E-cadherin-coated plates maintain pluripotent ES cells without colony formation. PLoS ONE.

[10] Nagaoka M, Si-Tayeb K, Akaike T, Duncan SA (2010). Culture of human pluripotent stem cells using completely defined conditions on a recombinant E-cadherin substratum. BMC Dev. Biol..

[11] Frixen UH (1991). E-cadherin-mediated cell-cell adhesion prevents invasiveness of human carcinoma cells. J. Cell Biol..

[12] Berx G (1995). E-cadherin is a tumour/invasion suppressor gene mutated in human lobular breast cancers. EMBO J..

[13] Janda E (2006). Raf plus TGFβ-dependent EMT is initiated by endocytosis and lysosomal degradation of E-cadherin. Oncogene.

[14] Sánchez-Tilló E (2010). ZEB1 represses E-cadherin and induces an EMT by recruiting the SWI/SNF chromatin-remodeling protein BRG1. Oncogene.

[15] Padmanaban V (2019). E-cadherin is required for metastasis in multiple models of breast cancer. Nature.

[16] Haque A (2011). The effect of recombinant E-cadherin substratum on the differentiation of endoderm-derived hepatocyte-like cells from embryonic stem cells. Biomaterials.

[17] Xu J (2013). hE-cadherin–Fc fusion protein coated surface enhances the adhesion and proliferation of human mesenchymal stem cells. Colloids Surf., B.

[18] Meng Q-Y, Akaike T (2013). Maintenance and induction of murine embryonic stem cell differentiation using E-cadherin-Fc substrata without colony formation. Front. Mater. Sci..

[19] Zhang Y (2016). Surface modification with E-cadherin fusion protein for mesenchymal stem cell culture. J. Mater. Chem. B.

[20] Cao L (2019). Construction of multicellular aggregate by E-cadherin coated microparticles enhancing the hepatic specific differentiation of mesenchymal stem cells. Acta Biomater..

[21] Yue X-S (2010). A fusion protein N-cadherin-Fc as an artificial extracellular matrix surface for maintenance of stem cell features. Biomaterials.

[22] Haque A, Yue X-S, Motazedian A, Tagawa Y-I, Akaike T (2012). Characterization and neural differentiation of mouse embryonic and induced pluripotent stem cells on cadherin-based substrata. Biomaterials.

[23] Haque A (2015). An engineered N-cadherin substrate for differentiation, survival, and selection of pluripotent stem cell-derived neural progenitors. PLoS ONE.

[24] Fujikake K (2018). Detachment of chain-forming neuroblasts by fyn-mediated control of cell–cell adhesion in the postnatal brain. J. Neurosci..

[25] Xu K (2016). Human VE-cadherin fusion protein as an artificial extracellular matrix enhancing the proliferation and differentiation functions of endothelial cell. Biomacromol.

[26] Jinnou H (2018). Radial glial fibers promote neuronal migration and functional recovery after neonatal brain injury. Cell Stem Cell.

[27] Pyne A, Thompson R, Leung C, Roy D, Hoogenboom BW (2014). Single-molecule reconstruction of oligonucleotide secondary structure by atomic force microscopy. Small.

[28] Harrison OJ (2011). The extracellular architecture of adherens junctions revealed by crystal structures of type i cadherins. Structure.

[29] Baumgartner W (2000). Cadherin interaction probed by atomic force microscopy. Proc. Natl. Acad. Sci..

[30] Brasch J (2011). Structure and Binding Mechanism of Vascular Endothelial Cadherin: A Divergent Classical Cadherin. J. Mol. Biol..

[31] Harrison OJ, Corps EM, Berge T, Kilshaw PJ (2005). The mechanism of cell adhesion by classical cadherins: the role of domain 1. J. Cell Sci..

[32] Yap AS, Brieher WM, Pruschy M, Gumbiner BM (1997). Lateral clustering of the adhesive ectodomain: a fundamental determinant of cadherin function. Curr. Biol..

[33] Norvell SM, Green KJ (1998). Contributions of extracellular and intracellular domains of full length and chimeric cadherin molecules to junction assembly in epithelial cells. J. Cell Sci..

[34] Pertz O (1999). A new crystal structure, Ca2+dependence and mutational analysis reveal molecular details of E-cadherin homoassociation. EMBO J..

[35] Niessen CM, Leckband D, Yap AS (2011). Tissue organization by cadherin adhesion molecules: dynamic molecular and cellular mechanisms of morphogenetic regulation. Physiol. Rev..

[36] Perez TD, Nelson WJ (2004). Cadherin adhesion: mechanisms and molecular interactions. Handb Exp Pharmacol.

[37] Perez, T. D. & Nelson, W. J. *Cell Adhesion*. 3–21 (Springer, 2004).

[38] Zhang Y, Sivasankar S, Nelson WJ, Chu S (2009). Resolving cadherin interactions and binding cooperativity at the single-molecule level. Proc. Natl. Acad. Sci..

[39] Sivasankar S, Zhang Y, Nelson WJ, Chu S (2009). Characterizing the initial encounter complex in cadherin adhesion. Structure.

[40] Wu Y, Vendome J, Shapiro L, Ben-Shaul A, Honig B (2011). Transforming binding affinities from three dimensions to two with application to cadherin clustering. Nature.

[41] Manibog K (2016). Molecular determinants of cadherin ideal bond formation: Conformation-dependent unbinding on a multidimensional landscape. Proc. Natl. Acad. Sci..

[42] Senoo A (2018). Inhibition of homophilic dimerization and disruption of cell adhesion by P-cadherin-specific small molecules from SPR-based assays. Chem. Commun..

[43] Hong S, Troyanovsky RB, Troyanovsky SM (2010). Spontaneous assembly and active disassembly balance adherens junction homeostasis. Proc. Natl. Acad. Sci..

[44] Lobner E (2017). Fcab-HER2 Interaction: a menage a trois: lessons from X-Ray and Solution Studies. Structure.

[45] Haque MA, Nagaoka M, Hexig B, Akaike T (2010). Artificial extracellular matrix for embryonic stem cell cultures: a new frontier of nanobiomaterials. Sci. Technol. Adv. Mater..

[46] Boggon TJ (2002). C-cadherin ectodomain structure and implications for cell adhesion mechanisms. Science.

[47] Pokutta S, Herrenknecht K, Kemler R, Engel J (1994). Conformational changes of the recombinant extracellular domain of E-cadherin upon calcium binding. Eur. J. Biochem..

[48] Tomschy A, Fauser C, Landwehr R, Engel J (1996). Homophilic adhesion of E-cadherin occurs by a co-operative two-step interaction of N-terminal domains. EMBO J..

[49] Häussinger D (2004). Proteolytic E-cadherin activation followed by solution NMR and X-ray crystallography. EMBO J..

[50] PyMOL. *The current version is 2.4. The free version is no longer available.*, https://pymol.org/ (2020).

[51] Vesenka J (1992). Substrate preparation for reliable imaging of DNA molecules with the scanning force microscope. Ultramicroscopy.

[52] Zenhausern F (1992). Scanning force microscopy and cryo-electron microscopy of tobacco mosaic virus as a test specimen. Ultramicroscopy.

[53] Edelman GM (1969). The covalent structure of an entire γG immunoglobulin molecule. Proc. Natl. Acad. Sci..

[54] Adamcik J, Berquand A, Mezzenga R (2011). Single-step direct measurement of amyloid fibrils stiffness by peak force quantitative nanomechanical atomic force microscopy. Appl. Phys. Lett..

[55] Heu C, Berquand A, Elie-Caille C, Nicod L (2012). Glyphosate-induced stiffening of HaCaT keratinocytes, a Peak Force Tapping study on living cells. J. Struct. Biol..

[56] Eghiaian F, Rigato A, Scheuring S (2015). Structural, mechanical, and dynamical variability of the actin cortex in living cells. Biophys. J ..

[57] Shibata-Seki T (2015). AFM characterization of chemically treated corneal cells. Anal. Bioanal. Chem..

[58] Israelachvili, J. N. in *Intermolecular and Surface Forces (Third Edition)* (ed Jacob N. Israelachvili) 291–340 (Academic Press, 2011).

[59] Ando T (2006). High-speed atomic force microscopy for studying the dynamic behavior of protein molecules at work. Jpn. J. Appl. Phys..

[60] Uchihashi T, Iino R, Ando T, Noji H (2011). High-speed atomic force microscopy reveals rotary catalysis of rotorless F1-ATPase. Science.

[61] Li J (2013). High-speed AFM for scanning the architecture of living cells. Nanoscale.

[62] Ando T (2018). High-speed atomic force microscopy and its future prospects. Biophys. Rev..

[63] Heath GR, Scheuring S (2019). Advances in high-speed atomic force microscopy (HS-AFM) reveal dynamics of transmembrane channels and transporters. Curr. Opin. Struct. Biol..

[64] Krieg M (2019). Atomic force microscopy-based mechanobiology. Nat. Rev. Phys..

