# Supplementary material for: Direct visualization of the extracellular binding structure of E-cadherins in liquid
Source: Sci Rep. 2020 Oct 12;10:17044. doi: 10.1038/s41598-020-72517-2 (PMC7552386; doi:10.1038/s41598-020-72517-2)
Supplement: Supplementary file 1 [file 41598_2020_72517_MOESM1_ESM.zip › Supplementary Information 0710.pdf]

Supplementary Information for

Direct Visualization of the extracellular binding structure of E-cadherins in liquid

Teiko Shibata-Seki[1], Masato Nagaoka[1], Mitsuaki Goto[1]*, Eiry Kobatake[2], Toshihiro Akaike[1]

1. Biomaterials Center for Regenerative Medical Engineering, Foundation for Advancement of International Science, 24-16 Kasuga, 3-chome, Tsukuba, Ibaraki 305-0821, Japan

2. School of Life Science and Technology, Tokyo Institute of Technology, G1-13, 4259, Nagatsuta, Midori-ku, Yokohama, Kanagawa 226-8502, Japan

*corresponding author, e-mail: goto@fais.or.jp

Supplementary data S1

Adhesive activity of E-cadherin using E-cad-Fc coated polystyrene beads.

Methods

Polystyrene bead coating

The standard polystyrene bead solution (IMMUTEX-Plain P2117, JSR life Science Corp., Japan) provided by JSR Corporation was placed in a 1 ml Eppendorf tube and centrifuged at 15,000 rpm for 30 minutes. The supernatant was discarded, the obtained pellet was dispersed in 1 ml of PBS solution without Ca^{2+}, and centrifuged under the same conditions. The supernatant was discarded, the obtained pellet was dispersed in 1 ml of PBS solution without Ca^{2+}, and used for the experiment. E-cad-Fc was prepared as a 5 µg/ml PBS solution without Ca^{2+}. One ml of E-cad-Fc solution was added to 100 µl of the above standard polystyrene bead solution, and coating was performed at 37° C. for 2 hours. This solution was placed in a 1 ml Eppendorf tube and centrifuged at 15, 000 rpm for 30 minutes. The supernatant was discarded, the obtained pellet was dispersed in 1 ml of PBS solution without Ca^{2+}, and centrifuged under the same conditions. The supernatant was discarded, the obtained pellet was dispersed in 1 ml of PBS solution without Ca^{2+}, and used for the experiment.

Dynamic light scattering (DLS) measurement

Dynamic light scattering (DLS) measurement was carried out using Otsuka Electronics FPAR-1000（Otsuka Electronics Co., Ltd. Japan). The particle size was measured by the cumulant average particle size and histogram average particle size (D50) (100 integrations). Five ml of PBS solution without Ca^{2+} was put in the measurement cell, 100 µl of each sample solution was added, and measurement was performed at 25° C to calculate the cumulant average particle diameter and histogram average particle diameter. Calcium Chloride solution was added just before the measurement so that the final concentration became the target concentration, and the solution was mixed well, and then the measurement was performed immediately.

Results

The results of DLS analysis of polystyrene beads coated with E-cad-Fc are as follows. Beads with a diameter of 121 nm were 148.3 (5.6) nm in a PBS solution without Ca^{2+}, beads with E-cad-Fc coating were 174.2 (35.8) nm in a PBS solution without Ca^{2+}, 329.5 (77. 3) nm in a PBS solution containing 2 mM Ca^{2+} (The data are mean (SD)). It is considered that the beads are adsorbed by the presence of Ca^{2+}.

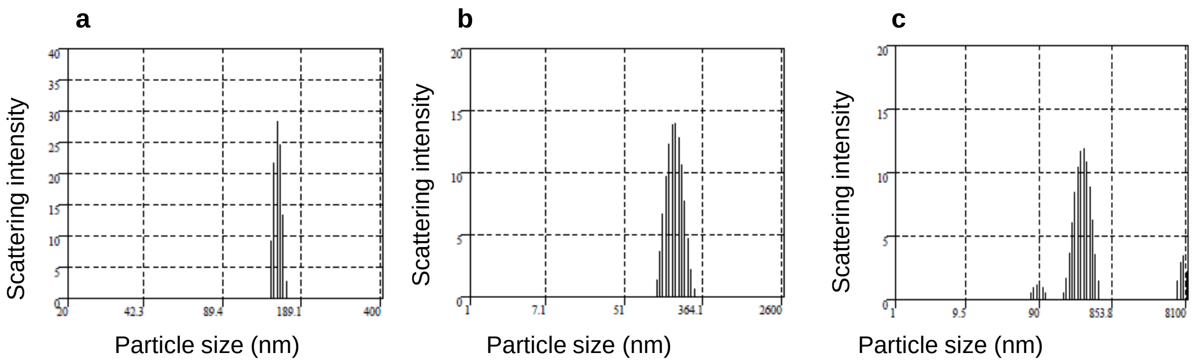

Figure S1. Particle size distributions from DLS **(a)** Polystyrene beads were 148.3 (5.6) nm in a PBS solution without Ca^{2+}. **(b)** Polystyrene beads with E-cad-Fc coating were 174.2 (35.8) nm in a PBS solution without Ca^{2+}. **(c)** Polystyrene beads with E-cad-Fc coating were 329.5 (77. 3) nm in a PBS solution containing 2mM Ca^{2+}. (The Data are mean (SD))

Supplementary Video S2.

3D structural model of IgG Fc.

Crystallographic structure model (PDB ID: 5JII) of IgG-Fc domain created with PyMOL. Hinge regions are not included in the model. The three-dimensional structure is shown in movie.

Ref.42 Lobner, E. *et al*. Fcab-HER2 Interaction: a menage a trois. Lessons from X-Ray and Solution Studies. *Structure* **25**, 878-889.e875, doi:10.1016/j.str.2017.04.014 (2017).

S3

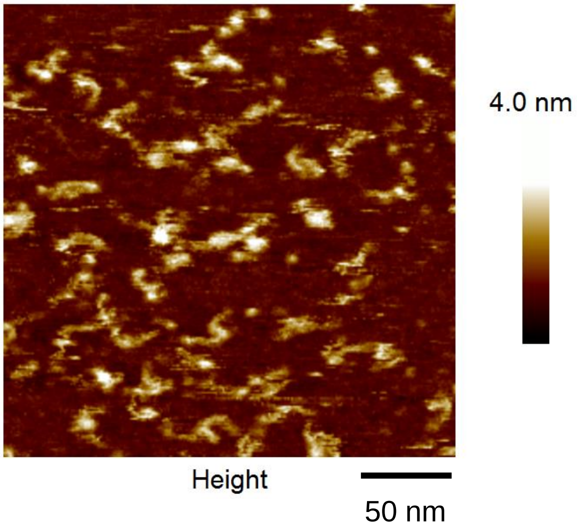

Supplementary Figure S3.

AFM images of E-cad-Fc molecules in a PBS solution containing 0.9 mM Ca^{2+}.

The samples were captured in 256 x 256 data points for 250 nm scan area with 1.3 Hz, the force-distance curves were recorded 20nm (PF amplitude of 10 nm) at a frequency of 2 kHz, using the standard probe for PFT measurements (SCANASYST-EFLUID+, Bruker Nano Inc., Goleta, CA, USA) with a spring constant of 0.7 Nm^{-1}, a nominal resonant frequency of 150 kHz in air, a nominal tip radius of 2 nm and reflective gold coating cantilever.

S4

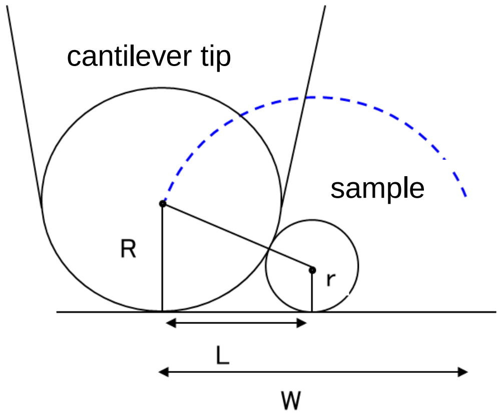

Supplementary Figure S4.

Convolution effect of AFM image.

An apparent width (W) becomes $W=4(Rr)^{1/2}$, where R is the radius of curvature of the cantilever tip and r is the radius of a sample which is assumed to have a circular cross section.

Ref.48 Vesenka, J. *et al.* Substrate preparation for reliable imaging of DNA molecules with the scanning force microscope. *Ultramicroscopy* **42-44**, 1243-1249, doi:https://doi.org/10.1016/0304-3991(92)90430-R (1992).

Ref.49 Zenhausern, F. *et al.* Scanning force microscopy and cryo-electron microscopy of tobacco mosaic virus as a test specimen. *Ultramicroscopy* **42-44**, 1168-1172, doi:https://doi.org/10.1016/0304-3991(92)90419-K (1992).
